# Comparison of Two Experimental Mouse Dry Eye Models through Inflammatory Gene Set Enrichment Analysis Based on a Multiplexed Transcriptomic Approach

**DOI:** 10.3390/ijms221910770

**Published:** 2021-10-05

**Authors:** Karima Kessal, Philippe Daull, Nicolas Cimbolini, Laurence Feraille, Sophie Grillo, Mylène Docquier, Christophe Baudouin, Françoise Brignole-Baudouin, Jean-Sébastien Garrigue

**Affiliations:** 1 INSERM UMR 968, CNRS UMR 7210, Institut de la Vision, IHU ForeSight, Sorbonne Université UM80, 75012 Paris, France; cbaudouin@15-20.fr (C.B.); fbaudouin@15-20.fr (F.B.-B.); 2 Centre Hospitalier National d’Ophtalmologie des Quinze-Vingts, INSERM-DGOS CIC1423, IHU FOReSight, 75012 Paris, France; 3 Centre Hospitalier National d’Ophtalmologie des Quinze-Vingts, Service 3, 75012 Paris, France; 4 SANTEN SAS, Ophthalmic Innovation Center, 91000 Evry-Couronnes, France; philippe.daull@santen.com (P.D.); Jean-Sebastien.Garrigue@santen.com (J.-S.G.); 5 Iris Pharma SAS, 06610 La Gaude, France; n.cimbolini@iris-pharma.com (N.C.); l.feraille@iris-pharma.com (L.F.); s.grillo@iris-pharma.com (S.G.); 6 iGE3 Genomics Platform, Figu Medical School, University of Geneva, CH-1211 Geneva, Switzerland; Mylene.Docquier@unige.ch; 7 Ambroise Paré, APHP, Department of Ophthalmology, University Paris Saclay, 92100 Boulogne, France; 8 Faculté de Pharmacie, Université de Paris, 75006 Paris, France

**Keywords:** dry eye mouse model, inflammatory signaling pathways, biomarkers

## Abstract

The goal of this study was to explore the specific signaling pathways related to inflammation in two experimental mouse dry eye (EDE) models. Female C57BL/6 mice housed for 10 days in a controlled desiccative environment were either treated with scopolamine (EDE-1; *n* = 18) or subjected to extraorbital lacrimal gland excision bilaterally (EDE-2; *n* = 10). Non-induced mice (*n* = 20) served as healthy controls. A corneal fluorescein staining (CFS) scoring was used at baseline through to day (D) 10 to evaluate epitheliopathy. At D10, corneas and conjunctivas were collected for multiplexed transcriptomic analysis with the NanoString^®^ mouse inflammatory CodeSet. Both EDE-1 and EDE-2 mice presented a change in corneal integrity, with a significant increase in CFS scores at D10. More gene transcripts were identified in EDE-2 compared with EDE-1 (116 vs. 96, respectively), and only a few were common to both models, 13 for the cornea and 6 for the conjunctiva. The gene functional annotation analysis revealed that the same inflammatory pathways were involved in both models. Comparative profiling of gene expression in the two EDE models leads to the identification of various targets and signaling pathways, which can be extrapolated to and confirmed in human disease.

## 1. Introduction

Dry eye disease (DED) is a multifactorial disease affecting the ocular surface and is defined by the loss of tear film homeostasis, resulting in destabilized tear film and hyperosmolarity, alterations in the corneal and conjunctival epithelia, inflammation, and neurosensory abnormalities [1]. Consequently, the quality of life of patients with DED symptoms can be significantly degraded [2,3].

Over the past few decades, numerous research studies have described modulation of inflammatory mediators in the tear film or ocular surface tissues, supporting the hypothesis that inflammation is one of the core mechanisms of DED [4,5,6,7,8] (Table 1).

Indeed, it has been well established that DED is an immune-mediated inflammatory disease of the lacrimal functional unit (LFU), which includes the cornea, conjunctiva, meibomian glands, lids, lacrimal glands, and sensory and motor nerves. Any alteration or stress to any of these tissues may contribute to the initiation of DED. The resulting cellular and functional dysregulation will exacerbate the signs and symptoms of DED through a self-sustained vicious cycle [7], leading to chronic DED [28,29]. However, the mechanisms explaining how and when tear film homeostasis is disrupted following local insult or inflammation of the ocular surface remain unclear. In parallel with clinical research, the development of in vivo experimental dry eye (EDE) models that mimic human DED [30,31] has allowed for the mechanistic exploration of the role played by the inflammatory/immune pathways in DED [31]. The commonly described models involve a reduction in tear production, either via pharmacological or surgical approaches. Pharmacological blockade of tear production by systemic diffusion of scopolamine and exposure to environmental desiccating stress [32] in mice are currently the most frequently used and have recently reached the status of the standard EDE model. The most recent EDE model of reduced tear production was achieved through lacrimal gland ablation [33,34]. In an attempt to highlight the potential application of these models to human disease, the major molecular findings and biological processes involved in both EDE models are summarized in Table 2. Hence, despite the anatomical differences between humans and mice and the experimental nature of these in vivo mouse EDE models, they represent suitable translational research tools. These observations reinforce the importance of studying DED mechanisms and exploring new therapeutic approaches.

The purpose of the present study was to compare the inflammatory mediators and signaling pathways involved in the cornea and conjunctiva in these two well-established murine EDE models. A multiplexed transcriptomic analysis was used to identify similarities and specificities of targets or signaling pathways in each model. The inflammatory profile of each model will be of significant interest in validating the targets and ensuring that the chosen model is fit for purpose.

## 2. Results

### 2.1. Induction of Dry Eye in Both Models

**Figure 1:**
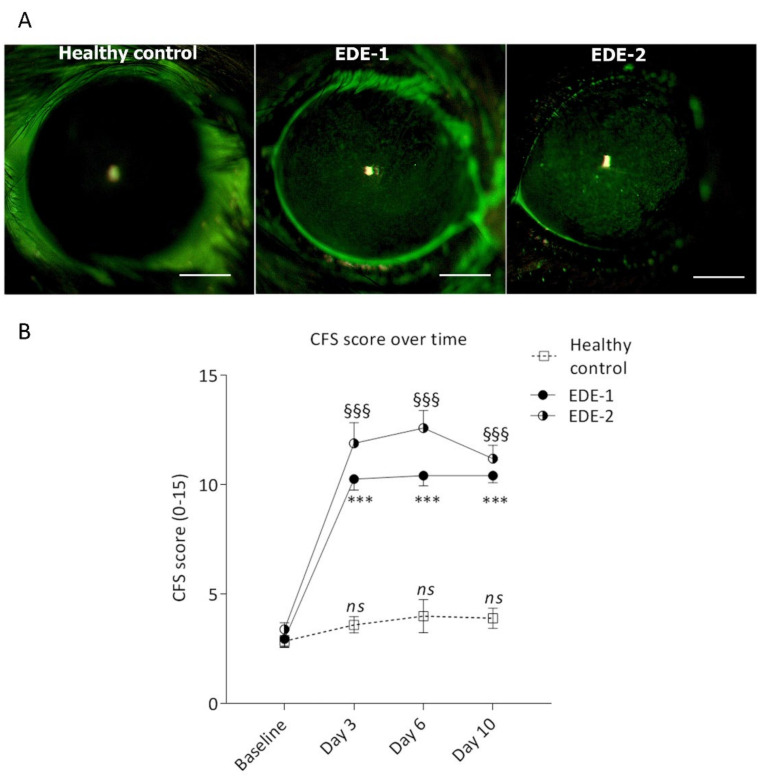
Evaluation of corneal alterations in both mouse models: (**A**) Representative photographs of mouse cornea after fluorescein staining at day (D) 10 following induction of dry eye; (**B**) corneal fluorescein staining (CFS) scores over time at D3, D6, and D10. Data are expressed as the mean ± SEM, and one-way ANOVA comparisons using the Kruskal–Wallis test were performed in each group against baseline *** *p* < 0.0001, ^§§§^ *p* < 0.0001, respectively. EDE-1, mouse treated with scopolamine and placed in a controlled environment room; EDE-2, mouse following extraorbital lacrimal gland excision; CFS, corneal fluorescein staining.

Upon placement in a CER with a desiccative controlled environment, mice treated with scopolamine (EDE-1) or having undergone excision of their extraorbital lacrimal glands (EDE-2) were examined for corneal epitheliopathy via corneal fluorescein staining (CFS) (Figure 1A). Basal CFS scores showed no difference between the three groups, with a value of 3. Corneal epitheliopathy increased at D3, reaching 10.26 ± 0.5 and 11.9 ± 0.9 for EDE-1 and EDE-2, respectively, and a stable CFS score was observed for healthy controls at 2.85 ± 0.2. The increased CFS remained stable until D10, reaching 10.42 ± 0.3 and 11.2 ± 0.6 for EDE-1 and EDE-2, respectively (Figure 1B). There was no difference in CFS scores at D10 between the two models.

### 2.2. Cornea and Conjunctiva Differentially Expressed Genes (DEGs) in Both EDE Models

**Figure 2:**
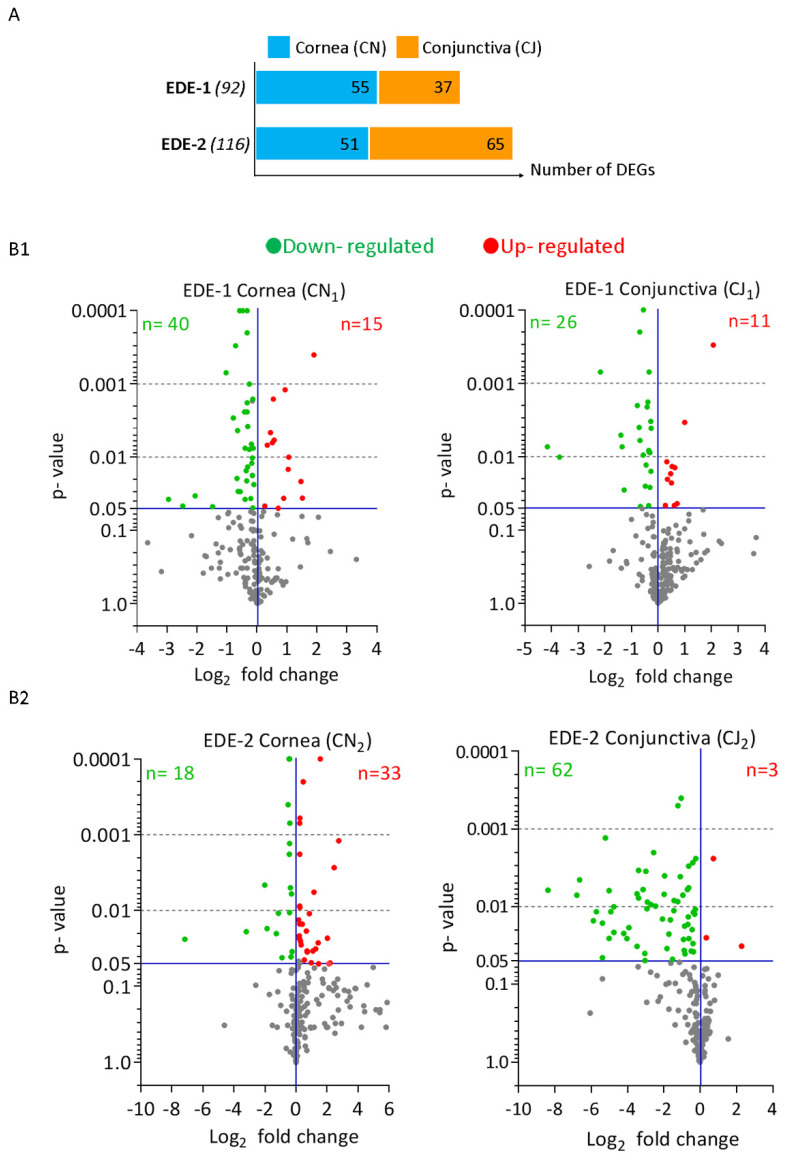
Differentially expressed genes (DEGs) in cornea and conjunctiva of both EDE mouse models: (**A**) Number and distribution of DEG in cornea and conjunctiva. (**B**) Volcano plot of DEGs between healthy controls and EDE-1 (**B1**) and EDE-2 (**B2**) depending on the tissue. *X*-axis and *y*-axis represent log_2_ fold-change difference between experimental model against healthy controls, respectively, and statistical significance as the *p*-value. Significantly up-regulated and down-regulated genes are indicated with red and green dots, while non-significant genes are shown as gray dots. The number of DEGs is indicated between brackets. (**B1**) DEGs distribution of EDE-1 in cornea (CN_1_) and conjunctiva (CJ_1_). (**B2**) DEGs distribution in EDE-2 in cornea (CN_2_) and conjunctiva (CJ_2_). CN, cornea; CJ, conjunctiva.

Among the 248 inflammatory-related genes present on the NanoString^®^ inflammatory CodeSet, 92 and 116 transcripts were significantly modulated during the progression of the DED in the cornea and conjunctiva for EDE-1 and EDE-2 models, respectively (Figure 2A). The numbers of DEGs were 55 and 51 in the cornea and 37 and 67 in the conjunctiva for EDE-1 and EDE-2, respectively. Figure 2B presents these data as a volcano plot to highlight up- and down-regulated DEGs according to their significance, with baseline levels in the cornea and conjunctiva. Several differences were observed between tissues and models.

In EDE-1, the cornea and conjunctiva presented more down-regulated than up-regulated transcripts (Figure 2B1); inversely, in EDE-2, the cornea presented more up-regulated transcripts than the conjunctiva (Figure 2B2). Indeed, in the EDE-1 cornea, among the 55 DEGs, 40 transcripts were down-regulated, while among the 51 DEGs in the EDE-2 cornea, only 18 were modulated. On the other hand, in the conjunctiva, both models showed more down-regulated than up-regulated genes, with more for EDE-2, with 62 transcripts compared with 26 for EDE-1. The list of DEGs in the cornea and conjunctiva for both EDE-1 and EDE-2 models, along with their fold changes relative to the healthy control group, are presented in Supplementary Tables S1 and S2.

### 2.3. Similarities and Specificities of DEGs in the Cornea and Conjunctiva in Both EDE Models

**Figure 3:**
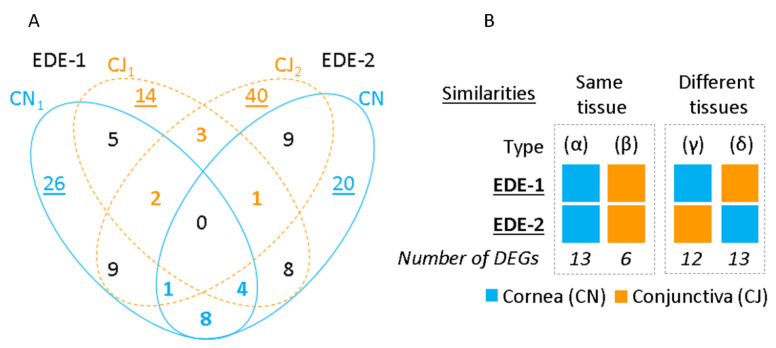
Tissue similarities and specificities between both EDE mouse models: (**A**) Venn diagram showing the overlap of DEGs between cornea and conjunctiva in each model. The colored and bold numbers highlight similarities within the same tissue, whereas underlined numbers indicate tissue specificity in both mouse models. (**B**) Four types of similarities between models according to tissue distribution are represented: within the same tissue as type (α) for cornea and type (β) for conjunctiva, and in different tissues as type (γ) and (δ) for cornea and conjunctiva of EDE-1 and EDE-2 respectively. Each EDE tissue is depicted as colored squares. The number of DEGs is represented for each type of group of similarities.

The Venn diagram in Figure 3A presents the number of DEG similarities and exclusivities between EDE models in the cornea and conjunctiva. In EDE-1, 26 of the 55 DEGs in the cornea, and 14 of the 37 DEGs in the conjunctiva, were exclusively modulated. Furthermore, in EDE-2, 20 of the 51 DEGs were unique to the cornea, and 40 of the 65 DEGs were exclusively associated with the conjunctiva. Upon exploring the similarities in expression between tissues in each model, 11 similar DEGs were found. However, none of them were common for both tissues and for both models. Upon exploring the similarities between the EDE-1 and EDE-2 models (Figure 3B), 13 and 6 DEGs were common to both models for the cornea (which we dubbed Type α) and conjunctiva (Type β), respectively. Furthermore, 12 DEGs were shared between the EDE-1 cornea and the EDE-2 conjunctiva (Type γ), while 13 DEGs were shared between the EDE-1 conjunctiva and the EDE-2 cornea (Type δ). Supplementary Table S3 presents the DEGs’ distribution among the four combinations generated by the comparison of these different tissues. In addition, Supplementary Figure S1 shows the up- and down-regulation of common genes within the same tissue.

### 2.4. Identification of Signaling Pathways and Molecular Profiles for Each EDE Model

Pathway analysis using the Reactome database [51] revealed that both models shared the same biological processes in response to ocular surface desiccative stress and corneal alterations. These targets identified in both models, in the cornea as well in the conjunctiva, highlighted the involvement of a common signaling pathway. Table 3 presents the predominant biological processes that involve the DEGs identified in the cornea and conjunctiva for both EDE models. Six major biological processes were identified through analysis of the DEGs profiles: (1) immune system, (2) signal transduction, (3) cellular responses to external stimuli, (4) gene expression (transcription), (5) programmed cell death, and (6) extracellular matrix organization.

Within these biological processes, the DEGs were distributed into various signaling pathways whose descriptions are presented in Table 4. Interestingly, while the identified pathways were the same for the cornea and conjunctiva and for the EDE-1 and EDE-2 models, the DEGs representative of these pathways were mostly different. Moreover, when DEGs similarities were observed, their modulations could be in the opposite direction (Supplementary Figure S1).

### 2.5. Description of the Main Representative Signaling Pathways: TLR, TNF, IFN, Programmed Cell Death, and Arachidonic Acid Metabolism

To highlight the molecular dysregulation similarities and specificities of each model and downstream effectors, we attempted to focus particularly on specific, relevant cellular cascades. These signaling pathways previously described in human as well as experimental dry eye models are described in Table 1 and Table 2, respectively.

**Figure 4:**
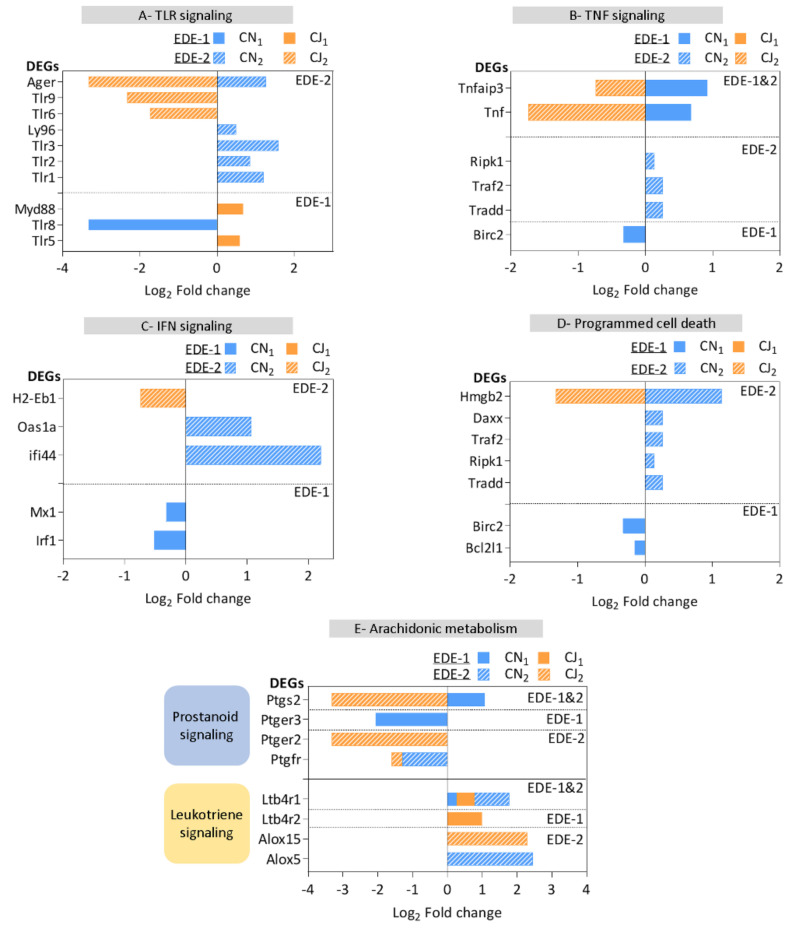
Main pathways involved in regulation of inflammatory status of both EDE models. Clustering of gene expression profiles highlights five signaling pathways: (**A**) Toll-like receptor (TLR) signaling; (**B**) tumor necrosis factor (TNF) signaling; (**C**) interferon (IFN) signaling; (**D**) programmed cell death (PCD); (**E**) arachidonic acid metabolism. Horizontal axis represents log2 fold-change difference between experimental models against healthy controls in each tissue: CN_1_ (cornea), CJ_1_ (conjunctiva) of EDE-1, and CN_2_ (cornea) and CJ_2_ (conjunctiva) of EDE-2. The hatched line separates EDE mice models DEGs’ specificities and similarities.

Toll-like receptors (TLRs), as a first-line non-specific immune response, were modulated in both EDE models (Figure 4A). Tlr1-2-3-6-9 genes were specifically modulated in the EDE-2 model, whereas Tlr 5-8 genes were modulated only in the EDE-1 model. Interestingly, the Ager gene was modulated exclusively in the EDE-2 model, with opposite modulations between the cornea and conjunctiva. These results suggest that different molecular mediators can be modulated in the TLR signaling pathways within the ocular surface upon the development of DED.

Interferon γ (IFNγ) and tumor necrosis factor α (TNFα) are well-described mediators of DED pathophysiology. Interestingly, the Tnf gene and its downstream effector Tnfaip3 gene were inversely regulated in both models (Figure 4B), supporting a role for their regulation in the disease. Moreover, second messengers in the IFN induction cascade, such as interferon stimulating genes (ISGs) (Figure 4C), appeared to be specifically modulated in the cornea of both models: Irf1 and Mx1 in the EDE-1 model and Ifi44 and Oas1a in the EDE-2 model. These results also highlight a corneal specificity for ISGs transcription.

Programmed cell death (PCD) and associated pathway modulations are presented in Figure 4D. As for ISGs, the corresponding DEGs were modulated specifically in the cornea of both models. Moreover, these cellular effectors were modulated mainly in EDE-2, with an up-regulation of Tradd, Ripk1, Traf2, Daxx, and Hmgb2 and down-regulation of only Bcl2l1 and Birc2 in EDE-1.

Finally, the DEGs associated with the arachidonic acid metabolism pathway (Figure 4E) pinpoint the involvement of the leukotriene and prostanoid pathways in both models. In the leukotriene signaling pathway, two leukotriene receptors, Ltb4r1 and Ltb4r2, appeared to be up-regulated in both models, whereas a specificity was observed for EDE-2 with the Alox5 and Alox15 genes of the two main enzymes in this pathway. Although Ltb4r1 modulation was similar in both models, it showed greater expression in EDE-2 corneas compared with EDE-1 corneas, with a fold change of 2 vs. 1.2, respectively. Nevertheless, Ltb4r2 up-regulation was associated only with the conjunctiva in EDE-1. Additionally, a prostanoid signaling pathway was described, with significant regulation of Ptgfr, ptger2, ptger3, and ptgs2. Prostanoid ligand receptors were down-regulated in both models; Ptgfr (FP) and Ptger2 (E2) were specifically associated with the EDE-2model, while Ptger3 (E3) was specifically down-regulated in the EDE-1 model. Interestingly, Ptgs2 (Cox2), a key enzyme for prostaglandin biosynthesis, was shared between models but in a mirrored regulation, with a significant decrease in EDE-2.

## 3. Discussion

The goal of this study was to assess the signaling pathways involved in the cornea and conjunctiva of two commonly used experimental mouse dry eye (EDE) models. Both models are characterized by a reduction in tear secretion, either through muscarinic receptor blockade (EDE-1) or extraorbital lacrimal gland excision (EDE-2), both in combination with exposure to a desiccative environment. We investigated the differential effects of these models on the pathogenesis of EDE disease. For this purpose, we employed a targeted multiplexed transcriptomic profiling approach, using Nanostring^®^ technology to identify targets known to be involved in specific inflammatory-related pathways and to describe similarities and exclusivities between these two models. Despite differences between human and mouse physiology, murine models have contributed greatly to the investigation of the major processes involved in dry eye. EDE models, especially in mice, represent useful tools in studying the finest regulation in inflammatory processes [31] and understanding the initiation of the inflammatory cascade that generates innate and adaptive immune responses [33]. As expected, the inhibition or loss of tear production from the lacrimal glands induced an alteration in corneal integrity in both models. Additionally, several DEGs were identified within the cornea and conjunctiva of both EDE mouse models. Likewise, our results highlighted the involvement of several signaling pathways in response to harmful events, consistent with previously described findings in both models (Table 2). Thus, the comparison of the human and mouse molecular mediators presented in Table 1 and Table 2, respectively, and our present data confirm that both EDE-1 and EDE-2 models are relevant for the exploration of inflammatory pathways associated with dry eye. The inflammatory profile of each EDE mice model and cell signaling network will be helpful to enrich the panel of biomarker candidates already described in human disease (Table 1), as these new inflammatory effectors could be an indication of the disease stage from the first steps of cellular dysregulation and regulation loops. In addition, thanks to the control and monitoring of these first steps, with defined stimuli using in vivo models, we are able to identify critical nodes in signaling pathways and important mediators in cellular responses in human pathologies. The role of the positive or negative regulation could also be a junction for potential crosstalk with other signaling systems.

These inflammatory pathways coordinate several biological events involving TLRs, cytokines, and apoptotic cellular events, among others [31]. Indeed, inflammation is a natural response and remains the first line of defense against microbial, toxic, or mechanical insults. It is a carefully orchestrated response of the organism to tissue aggression, damage, and changes in homeostasis. Likewise, the inflammatory response depends on the balance between pro- and anti-inflammatory signals. However, when that equilibrium is disturbed, a more widespread inflammatory response may take place [52].

Overall, in this detailed comparison of targets by examining the cornea and the conjunctiva, we demonstrated that variations in gene expression between two models exist. These differences may explain the mode of establishment and regulation of the clinical sign as well as its degree of severity. Interestingly, even when similar biological processes were involved in both models, such as immune responses or apoptosis, the molecular mediators and their regulation appeared to be highly different. This finding suggests a specific tissue regulation of cellular signaling information in each model. These complex networks are controlled by the interplay between various events, which restrict and terminate activation and inflammation to prevent the occurrence of inflammatory disease.

Indeed, the comparison of the different EDE tissues showed only 13 and 6 common transcripts in the cornea and conjunctiva, respectively. This low tissue similarity involves a majority of downstream targets of several signaling processes such as Hdac4, Rhoa, Gnas, Mapk3, Mapk3k1, and Mafg for the cornea, and Smad7, Nr3c1, and Tcf4 for the conjunctiva. These non-specific targets are implicated in protein metabolism, the signal from external stimuli, homeostasis, or gene expression. Some of these could represent interesting targets for pharmacological development, as in the case of histone deacetylase inhibitor (HDACi) as a potential anti-inflammatory agent [53]. Indeed, a therapeutic approach through inhibition of this enzyme induces the regulation of a variety of immunomodulatory transcripts in a dry eye mouse model [54]. While several targets offer new information on the triggering factors in the development of aberrant and/or persistent inflammation as well as in immune dysregulation, particular interest is being generated by specific signaling pathways, particularly those known to be involved in human disease and associated with the initiation and progression of inflammation.

The top five signaling pathways that have been considered are TLR, IFN, TNF, programmed cell death, and eicosanoid signaling. Toll-like receptors (TLRs), members of the highly conserved glycoprotein pattern recognition receptors (PRRs), have been extensively described in inflammation and defense processes. TLRs trigger inflammation via recognition of conserved motifs on pathogen-associated molecular patterns (PAMPs) from microbes and/or via damage-danger-associated molecular patterns (DAMPs) from damaged cells [55,56]. In addition, TLR activation triggers a complex signal transduction cascade that induces the production of inflammatory cytokines and co-stimulatory molecules, thus initiating innate and adaptive immunity [57,58]. Their involvement in various ocular surface diseases has also been widely described [19]. Indeed, modulation of TLR expression occurring in dry eye could play an important role in ocular surface susceptibility to inflammation and infection [20,59]. In our study, EDE-2 showed a higher number of differentially expressed TLR genes than EDE-1, suggesting more harmful events implicating the first line of defense. Nevertheless, increased TLR gene expression may enhance pathogen recognition but may also lead to inappropriate and exacerbated inflammatory responses, thereby contributing to disease processes such as DED or ocular allergy [60]. Likewise, the EDE-2 model also presented specificity in Ager (advanced glycosylation end-product specific receptor) with regulation and modulation in both the cornea and conjunctiva. This receptor, also known as RAGE, is an important effector of DAMPs. DAMPs, also known as alarmins or endogenous ligands, are indicative of tissue trauma [61]. It may involve intracellular components of ruptured cells, such as nucleic acids, extracellular DNA (eDNA), free fatty acids, or extracellular matrix (ECM) breakdown products [62]. Interestingly, Tlr1-2-9, known to recognize DAMPs [61], were significantly modulated in EDE-2, suggesting more significant tissue alteration through damaged or dying cells in this model.

We also noted that the response mediated by TNF and IFN was predominantly observed in the cornea. This might be explained by the barrier role of the cornea in defense against harmful external events. Interestingly, four ISGs, specifically regulated in the cornea in each model, have been previously described in conjunctival cells of dry eye patients [15]. Indeed, Irf1/Mx1 and Ifi44/Oas1a were modulated in EDE-1 and EDE-2, respectively. This aspect could be further examined in order to assess the relevance of this expression in relation to the etiology of the condition and involvement of IFN-I and IFN-II responses. Concerning TNF downstream signaling, the EDE models displayed an opposite modulation, which could be explained by different timing in the regulation of the inflammatory response. Indeed, Tnfaip3, an endogenous negative regulator of NF-kappa B signaling [63,64], was up-regulated in EDE-1 compared with EDE-2, suggesting a second level of loop regulation.

Among biological processes known to be involved in DED, apoptosis is a critical step in inflammatory responses [65]. Under normal circumstances, moderate inflammatory reactions and cell death are beneficial. However, excessive inflammation and abnormal activation of the cell death pathway often result in harmful consequences, finally leading to the pathogenesis of various human diseases. Likewise, the higher number of DEGs related to cell death in EDE-2, such as Tradd, Ripk1, Traf2, and Hmgb2, compared with the EDE-1 model, suggests a robust apoptotic response potentially associated with more chronic DED with a longer duration of DED symptoms [33,34]. An interesting target, such as Daxx, even though poorly described, might deserve further exploration for a better understanding of the regulation of apoptosis [66] or cell survival [67].

Finally, arachidonic acid metabolism with eicosanoid signaling, involving a family of proinflammatory lipid mediators, such as leukotrienes (LTs) and prostaglandins (PGs), was significantly modulated in both EDE models. Indeed, LTs and PGs, among others, are known to modulate immune responses. Interestingly, receptors of chemotactic eicosanoids were significantly up-regulated in both models. Ltb4r1, a high-affinity leukotriene receptor [68], was up-regulated in both models. Nevertheless, Ltb4r2 [69], a low-affinity receptor, was limited to EDE-1. These receptors act as signal relay molecules during neutrophil chemotaxis [70] and are required, as is integrin, for neutrophil swarms [71]. Additionally, two lipoxygenases (ALOXs) involved in lipid peroxidation, 5-lipoxygenase (Alox5), and arachidonate 15-lipoxygenase (Alox15), were specifically modulated in the EDE-2 model. ALOXs provide the largest contribution to the generation of lipid peroxides, preferentially through oxidized polyunsaturated fatty acids (PUFAs) [72]. ALOXs orchestrate the clearance of apoptotic cells and maintain immune tolerance [73]. Additionally, excessive lipid peroxidation suggests the induction of multiple patterns of cell death, including apoptosis, pyroptosis, and ferroptosis [72,74].

The molecular examination of these two ocular surface tissues, the cornea and the conjunctiva, showed clear differences, which could be explained by their specific anatomical features and their different cellular composition. This could also participate in the powerful link that exists in the homeostasis of these two tissues. Indeed, the cornea and conjunctiva, both consisting of stratified epithelium, serve as barriers to various insults. The conjunctiva, as opposed to the cornea, possesses a high density of soluble mucin-producing goblet cells, resident immune cells, and blood vessels [75,76]. The corneal epithelium includes corneal nerves, keratocytes, and very few resident immune cells, involving different cell–cell communication and paracrine effects.

Although further investigations remain necessary to understand the molecular differences between both EDE-1 and EDE-2, these mouse models involve quite different pathophysiological mechanisms. Indeed, as the two models affect different targets within similar signaling pathways, it would be interesting to evaluate the kinetics of expression of these targets to identify the initiating regulators and to follow disease progression. Several challenging investigations remain necessary to understand the dynamic interactions between targets and cells so as to enable a description of the vicious molecular circle that could be induced during the establishment of deleterious, non-reversible phenotypic effects.

## 4. Materials and Methods

### 4.1. Animals

Forty-nine C57BL/6 female mice aged from 6 to 9 weeks (Charles River Laboratories, Saint-Germain-Nuelles, France) were used. All animals were treated according to Directive 2010/63/UE of the European Convention for the Protection of Vertebrate Animals used for Experimental and Other Scientific Purposes as well as the Association for Research in Vision and Ophthalmology (ARVO) statement for the Use of Animals in Ophthalmic and Vision Research. The experimental protocol was in accordance with European Committee directives. The mice were housed at a constant temperature (22 ± 2 °C), controlled relative humidity (55 ± 10%), and in a light-controlled environment (lights on from 7 AM to 7 PM) with ad libitum access to food and water.

### 4.2. Controlled Environment Combined with Scopolamine EDE Model (EDE-1)

Nineteen female mice were placed in a controlled environment room (CER) for 10 days (temperature: 22 ± 2 °C; relative humidity <25%; airflow: 15 L/min) and were treated with a scopolamine transdermal patch applied every 48 h (0.5 mg/72h, Scopoderm^®^ TTS, Novartis, Reuil-Malmaison, France) starting from day 0 and following a procedure previously described [32,77]. They received no other treatment, and the ocular surface was monitored over a period of 10 days, with corneal fluorescein staining (CFS) at days 3, 6, and 10. At the end of the experimental procedure, the animals were euthanized, and the corneas and conjunctivas were sampled for further analysis.

### 4.3. Controlled Environment Combined with Extraorbital Lacrimal Gland Excision EDE Model (EDE-2)

Ten female mice were subjected to bilateral extraorbital lacrimal gland (ELG) excision. Briefly, after administration of analgesic and anesthesia, the ELGs were excised bilaterally. Following the surgical procedure, the mice were placed in a CER for 10 days (temperature: 22 ± 2 °C; relative humidity <25%; airflow: 15 L/min). These mice were examined over a period of 10 days according to the same protocol as that used for the EDE-1 model.

### 4.4. Healthy Controls

Aged-matched healthy C57BL/6 female mice (*n* = 20) were used as healthy controls. They were housed in standard housing conditions. The CFS scores were evaluated at the same time points as the induced mice and according to the protocol described below. They were then euthanized, and their corneas and conjunctivas were collected according to the protocol used for the EDE-1 and EDE-2 mice.

### 4.5. Evaluation of Corneal Changes

Corneal fluorescein staining was performed before dry eye induction (baseline) and during the experiment at days 3, 6, and 10, according to a previously published protocol [77,78]. Briefly, 0.5 µL of 0.5% fluorescein sodium solution (Fluoresceine Faure, 0.4 mL unit dose vials, Novartis Pharma SAS, France) was instilled in both eyes. The corneas were examined bilaterally using a biomicroscope with light passing through a cobalt blue filter. The stained area was assessed and graded by the grading system of the NEI/Industry Workshop guidelines [79]. The CFS score was evaluated by dividing the cornea into five zones, with each zone scored on a 0–3 scale, for a maximum total score of 15.

### 4.6. Tissue Sampling and Total RNA Isolation

The healthy control, EDE-1, and EDE-2 mice were euthanized at the conclusion of the experiment on day 10 via an intraperitoneal injection of overdosed pentobarbital, as recommended for euthanasia by the European authorities (French decree no. 2013–118) [80].

Immediately after euthanasia, the cornea and conjunctiva of one eye were collected and snap frozen. Both eyes were collected from healthy controls to increase available material for the baseline state. Samples were stored at −80 °C for further total RNA extraction and subsequent gene expression analysis. Total RNA was extracted from corneas and conjunctivae according to the manufacturer’s protocol using an RNA-XS kit from Macherey-Nagel (Macherey-Nagel, Hoerdt, France). Total RNA yield and integrity were assessed with an Agilent 2100 bioanalyzer (Agilent Technologies, Wilmington, DE, USA). RNAs with an RNA integrity number (RIN) greater than seven were used for analysis. As the purpose of this workflow was based on the evaluation of samples from each mouse and according to the available quantity and integrity of the RNA, six groups of RNA samples were obtained: healthy control cornea (*n* = 32) and healthy control conjunctiva (*n* = 20), EDE-1 cornea (*n* = 18) and EDE-1 conjunctiva (*n* = 9), and EDE-2 cornea (*n* = 10) and EDE-2 conjunctiva (*n* = 10).

### 4.7. Multiplexed mRNA Quantification using NanoString^®^ nCounter Assay

The inflammatory transcriptomic profiles were measured in the whole cornea and conjunctiva from all mice through a multiplexed hybridization assay and specific fluorescent barcode probes [81]. Gene expression was measured with the nCounter^®^ Mouse Inflammatory panel (NanoString Technologies, Seattle, WA, USA) on the NanoString nCounter Analysis System (NanoString Technologies, Seattle, WA, USA). The CodeSet used consists of biotinylated capture probes and reporter probes attached to color barcode tags for the 248 mouse genes related to inflammation and 6 internal reference genes (CLTC, GAPDH, GUSB, HPRT, PGK1, and TUBB5). Briefly, purified RNAs were diluted in nuclease-free water to 20 ng/μL, for a final assay dose of 100 ng. Samples were incubated 16–22 h at 65 °C as per the manufacturer’s standard protocol to ensure hybridization with reporter and capture probes. After hybridization, the samples were processed in the PrepStation and counted in the digital analyzer. The mRNA copy numbers were normalized against the internal reference genes, and the mean copy number per group was determined and used for comparison. The mRNA counts acquired after normalization were expressed as means of fold changes relative to control tissues of healthy mice to compare the effects of activation responses between tissues and models.

### 4.8. Functional Annotation and Signaling Pathways Network Analysis

To annotate and validate cross-talks between differentially expressed genes (DEGs), for functional annotation and pathway analysis, we used two popular and well-referenced annotation databases [82]: The Kyoto Encyclopedia of Genes and Genomes (KEGG) [83] https://www.genome.jp/kegg/pathway.html (accessed on 1 January 2020) and Reactome databases [51]. These pathway analyses were enriched with the Graphiteweb tool http://graphiteweb.bio.unipd.it/ (accessed on 1 January 2020) for network visualization [84].

### 4.9. Statistical Analysis

The statistical analysis and figure presentation were performed using GraphPad Prism 7.0 software (GraphPad Software; Carlsbad, CA, USA). CFS data were compared with Tukey’s multiple comparisons test. Statistical significance was set at a *p*-value of 0.05. Results are presented as mean ± SD. Gene expression data were log-transformed, and an unpaired *t*-test with unequal variance was then used to assess the significance (*p* < 0.05) in the fold changes between healthy and EDE-1 or EDE-2 mice.

## 5. Conclusions

Ocular desiccating stress results in an inflammatory response, with a downstream modulation of various intracellular signaling elements depending on pharmacological blockade or extraorbital lacrimal gland excision according to each mouse EDE model. The resulting molecular network involves coordinated regulation and extensive crosstalk between several intracellular signaling pathways, controlling the dysregulated processes of the pathology.

Transcript comparison between the two experimental dry eye mouse models identified a substantial number of targets involved in various signaling pathways. Description of this molecular mapping offers a wide range of molecular tools to explore the mechanisms of disease initiation and progression. This should bring new perspectives to highlight the interaction and regulatory events orchestrating biological processes in the inflammatory response in dry eye disease. Moreover, each mouse model represents a powerful, rational tool for the investigation of pathophysiology and biomarkers, which should prove useful in selecting the appropriate models for each future research objective.

Finally, extrapolation from mouse models to human disease remains an important method of identifying new therapeutic targets and improving knowledge of the etiologies of DED as a multifactorial disease.

## Figures and Tables

**Table 1:** Summary of current biological processes and molecular mediators involved in human dry eye disease.

Biological Processes	Signaling Pathways and Cell Activations	Dry Eye Disease (Human)
Immune system	*Cytokines*	IL-1β, IFNγ, TNFα, IL-6, IL-17A, IL-10, CCL2, TGFβ [9,10,11,12,13]
	*Chemokines*	CXCL9, 10, 11, CXCR3 [9,14,15,16] CCR4, CCR5 [17,18]
	*Toll-like receptors (TLRs)*	TLR4, TLR5, TLR9 [19,20]
	*Cellular infiltration*	HLA DR, CD4, CD8, CD3, CD11a [15,16,21,22,23,24]
	*Dendritic cell (DC) maturation*	HLA DR [21] morphological and density changes in DCs in cornea [25]
Signal transduction	*MAPK activation*	MK2, MAP2K6, MAPK8 [15,16,26]
Programmed cell death (PCD)	*Epithelial apoptosis*	CD40, CD40L, decrease (↘) cellular viability [15,16,25,27]
Extracellular matrix (ECM)	*MMPs*	MMP-9 [26]

**Table 2:** Summary of current biological processes and molecular mediators involved in two experimental dry eye mouse models.

Biological Processes	Signaling Pathways and Cell Activations	Pharmacological Model (Controlled Environment and Scopolamine)	Surgical Models (Controlled Environment and Lacrimal Gland Excision/Ablation)
Immune system	*Cytokines*	IL-1(α/β), IFNγ TNFα, IL-6; IL-18 [32,35,36,37] IL-17 A, IL-17 R, IL23, IL-23R, IL-22, TGFβ1, TGFβ2 [38]	IL-1β; TNFα [33]; IFNγ, IL-17 [39]
	*Chemokines*	CXCL9, CXCL10; CXCR3; CCL3, CCL4, CCL5; CCR5 [40]	
	*Toll-like receptors (TLRs)*	TLR2, TLR4, TLR9 [41]	
	*Cellular infiltration*	Increase (↗) CD4^+^/CD8^+^ in cornea and conjunctiva [38,42,43]	Increase (↗) neutrophils in cornea and conjunctiva [34]
	*Dendritic cell maturation*	APC development [44]	Increase (↗) CD45^+^ CD11b^+^ in cornea [45]
Signal transduction	*MAPK activation*	Activation of JNK1/2, ERK1/2, and p38 MAPKs [46], MK2 [47]	
Programmed cell death (PCD)	*Epithelial apoptosis*	Increase apoptosis (↗) in cornea and conjunctiva, Caspase-3-8 [48,49]	Increase apoptosis (↗) in corneal epithelial cells [45]
Extracellular matrix (ECM)	*MMPs*	MMP1-3-9- 10 [50]	MMP-9 [33]

**Table 3:** Overview of biological processes and current targets involved in both human disease and experimental mouse models.

	Differentially Expressed Genes (DEGs)							
	EDE-1				EDE-2			
	CN_1_		CJ_1_		CN_2_		CJ_2_	
	*Nb.*	(%)	*Nb.*	(%)	*Nb.*	(%)	*Nb.*	(%)
Biological processes								
Immune system	31	(56.4)	12	(32.4)	28	(54.9)	30	(46.2)
Signal transduction	21	(38.2)	19	(51.4)	17	(33.3)	31	(47.7)
Cellular responses to external stimuli	6	(10.9)	3	(8.1)	5	(9.8)	4	(6.2)
Gene expression (transcription)	6	(10.9)	5	(13.5)	3	(5.9)	3	(4.6)
Programmed cell death (PCD)	3	(5.5)	0	(0)	5	(9.8)	2	(3.1)
Extracellular matrix organization (ECM)	1	(1.8)	4	(10.8)	1	(2)	3	(4.6)
Total DEGs	**55**		**37**		**51**		**65**	

**Table 4:** Comparison of molecular targets involved in EDE. Summary of differentially expressed genes in corresponding signaling pathways in both experimental mouse models.

			EDE-1		EDE-2	
Biological Processes	Principal Pathways	Secondary Pathways	Cornea (CN_1_)	Conjunctiva (CJ_1_)	Cornea (CN_2_)	Conjunctiva (CJ_2_)
Immune System	Innate Immune response	Toll-like receptor cascades	*Tlr8; Ripk2; Nod1;Jun*	*Tlr5;Myd88; Nfkb1; Rps6ka5*	*Tlr1;Tlr2;Tlr3;Ly96; Rps6ka5; Ager*	*Tlr6; Tlr9;Ager;Nod2*
		Complement cascades	*C1qa; C1qb; C1s; C1ra*	*Cfd*	*C2; C4a;C1s;C3ar1*	*Cfb; C3;C2; Masp1; Hc*
		Antimicrobial peptides	*/*	*/*	*Tlr1; Tlr2*	*Cd4*
		Neutrophil degranulation	*Cxcl1; Rhoa*	*Cfd; Nfkb1*	*Tlr2; C3ar1; Arg1;Alox5; Nfkb1; Rhoa*	*Cxcl1; Cxcr1; Cxcl2; Cxcr2; C3; Tyrobp; Mmp9*
		NLR signaling pathway	*Nod1;Ripk2;Tnfaip3;Birc2; Bcl2l1*	*/*	*Nod1*	*Nod2; Nlrp3; Tnfaip3*
		NLR inflammasome	*Nod1*	*/*	*Nod1*	*Nod2; Nlrp3*
		DAP12 interactions	*Grb2; Shc1; Hras1*	*Grb2*	*/*	*Trem2; Tyrobp*
		C-type lectin receptors (CLRs)	*Hras1; Relb*	*Nfkb1;Rps6ka5; Raf1; Relb*	*Nfkb1; Rps6ka5*	*Il1b*
	Adaptative immune system	TCR signaling	*Ripk2*	*Nfkb1*	*Nfkb1*	*H2-Eb1; Cd4*
		Costimulation by the CD28 family	*Grb2*	*Grb2*	*/*	*H2-Eb1; Cd4*
Immune System *(Continued)*		Signaling by the B cell receptor (BCR)	*Grb2; Hras1;*	*Grb2; Nfkb1;*	*Nfkb1; Prkcb*	*Prkcb*
		MHC class II antigen presentation	*/*	*/*	*/*	*H2-Eb1*
		Interaction between lymphoid and non-lymphoid cells	*Cd40*	*/*	*/*	*Cd40; C3; Trem2*
		TNF signaling	*Tnf; Tnfaip3; Twist 2*		*Twist 2*	*Tnf; Tnfaip3*
		TNFR2 non-canonical NF-kB pathway	*Cd40; Tnf; Relb; Birc2*	*Relb*	*Ltb, Traf2*	*Ltb, Tnf, CD40; Tnfsf14*
		IFN signaling	*Irf1; Mx1*	*/*	*Oas1a; ifi44*	*H2-Eb1*
		FLT3 signaling	*Pdgfa; Hras1; Grb2; Shc1; Areg*	*Grb2; Rapgef2; Raf1*	*/*	*Pdgfa; Tlr9*
	Cytokines signaling	Signaling by interleukins	*Il1a;Il10rb;Il15;Tslp;Csf1;Grb2Ripk2;Nod1;Shc1;Jun*	*Il1rn; Grb2; Myd88; Nfkb1; Stat3; Rps6ka5*	*Il1rn;I Il1rap; Ltb; l10rb; IL18,Ager; Nod1; Traf2; Nfkb1;Rps6ka5; Creb1;*	*Il1b; Il23a;Il23r; Il22ra2;Cd4; IL18rap; Ager;Nod2*
		Chemokines	*Ccl2;Ccr2;Cxcl1*	*Cxcl5*	*Ccl11*	*Ccl2;Ccl7;Ccr2;Cxcl1;Cxcl5; Cxcl2;Cxcr2;Ccl3;Ccl8;Cxcr1; Cxcl10;Ccl19;Ccr7;Ccl21a; Cxcr4;Ccl17;Ccl22*
		Growth factor	*Pdgfa*	*Tgfb1;Tgfb2;Tgfb3; Smad7;Flt1*	*Tgfb3*	*Tgfb1;Tgfbr1;Smad7;Pdgfa*
Signal transduction	Cellular response to external stimuli	MAPK family signaling cascades	*Map3k1; Map2k6;Mapk14; Mapk3*	*Mapkapk2;Map2k6;Mknk1*	*Map3k1;Mapkapk2;Map2k4;Mapk3;Mapk1; Mknk1*	*Mapkapk2*
		Hypoxia	*/*	*/*	*Hif1a*	*Hif1a*
	Lipids signaling mediators	Leukotrien	*Ltb4r1*	*Ltb4r1;Ltb4r2*	*Ltb4r1; Alox5*	*Alox15*
		Prostanoid	*Ptger3; Ptgs2*	*/*	*Ptgfr*	*Ptgfr; Ptger2; Ptgs2*
PCD	Death receptor signaling	Apoptosis and necrosis	*Tnf;Birc2; Bcl2l1*	*/*	*Tradd; Traf2;Ripk1; Hmgb2;Daxx*	*Tnf; Hmgb2*
ECM	Matrix metalloproteinases	Signaling by TGF-beta family members		*Tgfb1;Tgfb2;Tgfb3; Mmp3*	*Tgfb3*	*Tgfb1;Tgfbr1; Mmp9*

## Data Availability

Not applicable.

## Supplementary Materials

The following are available online at https://www.mdpi.com/article/10.3390/ijms221910770/s1, Supplementary tables are represented (Tables S1–S3) and Supplementary Figure S1. Table S1: Differential expressed genes (DEGs) from EDE-1 in cornea and conjunctiva, Table S2: Differential expressed genes from EDE-2 in cornea and conjunctiva, Table S3: Molecular target distribution according to the type of similarities (α); (β); (γ); (δ), Figure S1: Pattern of common DEGs distribution between models within the same tissue.
