# Supplementary material for: Comparison of Two Experimental Mouse Dry Eye Models through Inflammatory Gene Set Enrichment Analysis Based on a Multiplexed Transcriptomic Approach
Source: Int J Mol Sci. 2021 Oct 5;22(19):10770. doi: 10.3390/ijms221910770 (PMC8509332; doi:10.3390/ijms221910770)

Supplementary Materials

Supplementary Table

Table S1. Differential expressed genes (DEGs) from EDE-1 in cornea and conjunctiva

(A) Up-regulated and down-regulated genes in cornea (**B**) Up-regulated and down-regulated genes in conjunctiva. Data are expressed as the mean ± SD, and t-test comparisons are performed against healthy controls at day 10.

**(A) Cornea**	**Healthy Controls** ***(n=32)***					**EDE-1** ***(n=18)***						
**Genes**	**Mean**	±	**SD**	**CV** *(%)*		**Mean**		**SD**	**CV** *(%)*	**FC**	**p-value**	
**Up-regulated (15)**												
Areg	957,4	±	435,9	45,5		3 581,1	±	2 520,9	70,4	**3,7**	*0,0004*	**
Ccl2	235,9	±	493,6	209,2		674,5	±	754,2	111,8	**2,9**	*0,0360*	*
Cxcl1	303,9	±	681,3	224,2		841,2	±	790,3	94,0	**2,8**	*0,0214*	*
Ptgs2	913,3	±	782,0	85,6		1 913,0	±	1 389,8	72,7	**2,1**	*0,0099*	***
Jun	4 278,9	±	2 510,3	58,7		8 825,0	±	6 944,0	78,7	**2,1**	*0,0146*	**
Tnfaip3	1 446,2	±	548,6	37,9		2 778,7	±	1 440,2	51,8	**1,9**	*0,0012*	***
Tslp	80,3	±	62,8	78,2		149,0	±	121,8	81,8	**1,9**	*0,0361*	**
Myc	2 945,4	±	563,1	19,1		5 286,1	±	1 694,8	32,1	**1,8**	*0,0000*	***
Tnf	256,6	±	230,4	89,8		421,8	±	295,0	69,9	**1,6**	*0,0495*	**
Relb	870,2	±	481,8	55,4		1 300,4	±	503,5	38,7	**1,5**	*0,0058*	***
Maff	1 201,1	±	400,5	33,3		1 754,1	±	593,1	33,8	**1,5**	*0,0016*	***
Il1a	1 266,0	±	351,3	27,7		1 810,2	±	716,2	39,6	**1,4**	*0,0063*	***
Cebpb	3 139,7	±	1 453,1	46,3		4 292,3	±	1 215,0	28,3	**1,4**	*0,0046*	***
Mafk	2 217,4	±	509,8	23,0		2 807,2	±	760,5	27,1	**1,3**	*0,0068*	***
Ltb4r1	613,3	±	220,5	36,0		734,7	±	187,9	25,6	**1,2**	*0,0462*	**
**Down-regulated (40)**												
Mapk14	5 774,5	±	715,2	12,4		5 342,4	±	566,7	10,6	**0,9**	*0,0235*	**
Pdgfa	4 524,5	±	474,9	10,5		4 140,9	±	451,3	10,9	**0,9**	*0,0075*	**
Shc1	6 790,5	±	611,4	9,0		6 203,4	±	571,9	9,2	**0,9**	*0,0016*	***
Bcl2l1	5 542,0	±	773,5	14,0		5 042,1	±	859,5	17,0	**0,9**	*0,0490*	*
Grb2	3 611,0	±	617,0	17,1		3 261,9	±	386,3	11,8	**0,9**	*0,0177*	*
Keap1	3 606,1	±	472,2	13,1		3 246,6	±	289,9	8,9	**0,9**	*0,0017*	***
Il10rb	4 093,9	±	615,9	15,0		3 679,3	±	463,8	12,6	**0,9**	*0,0102*	**
Hmgn1	43 764,9	±	7 808,4	17,8		39 317,8	±	4 951,6	12,6	**0,9**	*0,0176*	**
Rhoa	44 906,6	±	8 438,2	18,8		39 833,1	±	5 278,2	13,3	**0,9**	*0,0120*	**
Hdac4	1 880,1	±	303,3	16,1		1 662,2	±	230,3	13,9	**0,9**	*0,0066*	***
Hspb1	123 617,3	±	34 594,1	28,0		107 890,7	±	16 966,6	15,7	**0,9**	*0,0365*	*
Nr3c1	8 886,5	±	1 264,3	14,2		7 604,7	±	746,8	9,8	**0,9**	*0,0000*	***
Mapk3	16 377,9	±	3 692,9	22,5		13 769,8	±	1 456,4	10,6	**0,8**	*0,0010*	***
Bcl6	1 940,4	±	465,9	24,0		1 622,5	±	335,3	20,7	**0,8**	*0,0078*	**
Birc2	2 993,6	±	467,4	15,6		2 481,4	±	315,0	12,7	**0,8**	*0,0000*	***
Ripk2	926,0	±	272,3	29,4		753,0	±	199,4	26,5	**0,8**	*0,0135*	*
Gnas	25 026,5	±	8 289,1	33,1		20 227,4	±	2 272,2	11,2	**0,8**	*0,0038*	**
Nod1	2 097,7	±	450,4	21,5		1 686,6	±	264,9	15,7	**0,8**	*0,0002*	***
Map3k1	8 115,2	±	1 642,6	20,2		6 519,4	±	1 071,3	16,4	**0,8**	*0,0001*	***
Mx1	1 306,1	±	347,0	26,6		1 048,6	±	204,8	19,5	**0,8**	*0,0018*	**
Limk1	613,7	±	187,4	30,5		488,3	±	88,2	18,1	**0,8**	*0,0024*	**
Mef2a	1 048,8	±	352,7	33,6		833,3	±	274,9	33,0	**0,8**	*0,0209*	*

Cd40	894,4	±	332,1	37,1		695,2	±	224,5	32,3	**0,8**	*0,0152*	*
Mafg	924,7	±	366,2	39,6		704,0	±	192,2	27,3	**0,8**	*0,0075*	**
Hras1	354,9	±	171,3	48,3		269,9	±	109,0	40,4	**0,8**	*0,0374*	**
Irf1	26 603,8	±	8 291,2	31,2		19 912,7	±	6 256,8	31,4	**0,7**	*0,0024*	**
Map2k6	1 300,4	±	271,0	20,8		943,5	±	120,5	12,8	**0,7**	*0,0000*	***
Tcf4	6 942,0	±	2 119,9	30,5		4 988,8	±	1 012,2	20,3	**0,7**	*0,0001*	***
Csf1	1 033,0	±	629,9	61,0		705,9	±	398,0	56,4	**0,7**	*0,0293*	*
C1s	9 135,3	±	2 986,7	32,7		6 097,5	±	2 003,8	32,9	**0,7**	*0,0001*	***
C1qb	1 143,6	±	814,6	71,2		746,7	±	432,2	57,9	**0,7**	*0,0291*	*
Il15	322,9	±	179,3	55,5		206,2	±	96,3	46,7	**0,6**	*0,0043*	**
C1qa	961,3	±	682,3	71,0		602,9	±	364,6	60,5	**0,6**	*0,0194*	*
C1ra	600,9	±	294,7	49,0		363,7	±	132,7	36,5	**0,6**	*0,0003*	**
Twist2	677,2	±	426,3	62,9		390,0	±	220,4	56,5	**0,6**	*0,0029*	**
Plcb1	247,1	±	152,3	61,6		121,4	±	93,0	76,6	**0,5**	*0,0007*	**
Ccr2	95,5	±	123,7	129,6		34,3	±	87,1	254,2	**0,4**	*0,0471*	*
Ptger3	37,0	±	67,2	181,6		9,0	±	19,1	212,5	**0,2**	*0,0335*	*
Retnla	242,3	±	529,5	218,5		43,4	±	91,8	211,6	**0,2**	*0,0460*	*
Tlr8	26,1	±	57,9	221,4		3,4	±	10,1	298,7	**0,1**	*0,0373*	*

**(B) Conjunctiva**	**Healthy Controls** ***(n=20)***					**EDE-1** ***(n=9)***						
**Genes**	**Mean**		**SD**	**CV** *(%)*		**Mean**		**SD**	**CV** *(%)*	**FC**	**p-value**	
Up-regulated (11)												
Cxcl5	69,3	±	77,0	111,1		290,8	±	120,1	41,3	**4,2**	*0,0003*	***
Ltb4r2	194,4	±	141,9	73,0		387,0	±	138,6	35,8	**2,0**	*0,0034*	**
Myd88	2 555,6	±	1 137,4	44,5		4 184,4	±	1 985,9	47,5	**1,6**	*0,0435*	*
Tlr5	553,8	±	347,6	62,8		854,7	±	244,6	28,6	**1,5**	*0,0141*	*
Il1rn	606,9	±	422,4	69,6		923,1	±	342,4	37,1	**1,5**	*0,0460*	*
Ltb4r1	438,0	±	275,9	63,0		628,1	±	111,5	17,8	**1,4**	*0,0136*	*
Relb	1 836,8	±	878,3	47,8		2 592,7	±	702,0	27,1	**1,4**	*0,0228*	*
Mmp3	8 519,8	±	4 427,1	52,0		11 769,0	±	2 405,9	20,4	**1,4**	*0,0171*	*
Stat3	14 007,4	±	4 253,4	30,4		17 780,0	±	3 440,1	19,3	**1,3**	*0,0203*	*
Tgfb1	3 414,7	±	1 156,1	33,9		4 264,1	±	534,9	12,5	**1,2**	*0,0117*	*
Nfkb1	4 801,2	±	1 401,5	29,2		5 797,8	±	1 047,6	18,1	**1,2**	*0,0460*	*
Down-regulated (26)												
Grb2	5 086,2	±	438,4	8,6		4 247,3	±	641,8	15,1	**0,8**	*0,0041*	**
Rapgef2	4 370,9	±	799,0	18,3		3 613,0	±	666,5	18,4	**0,8**	*0,0158*	*
Hdac4	1 984,4	±	343,5	17,3		1 634,2	±	223,6	13,7	**0,8**	*0,0033*	**
Hspb1	28 244,0	±	7 141,6	25,3		22 853,4	±	4 814,8	21,1	**0,8**	*0,0262*	*
Tcf4	8 573,3	±	1 287,5	15,0		6 899,4	±	1 413,2	20,5	**0,8**	*0,0088*	**
Raf1	6 833,7	±	746,8	10,9		5 410,0	±	847,5	15,7	**0,8**	*0,0007*	***
Gnas	28 572,2	±	8 624,3	30,2		22 567,5	±	2 574,4	11,4	**0,8**	*0,0087*	**
Smad7	1 030,5	±	226,4	22,0		806,2	±	266,5	33,1	**0,8**	*0,0464*	*
Mapkapk2	8 314,4	±	2 559,0	30,8		6 490,9	±	837,7	12,9	**0,8**	*0,0082*	**
Nr3c1	7 494,3	±	1 152,7	15,4		5 735,1	±	1 165,3	20,3	**0,8**	*0,0018*	**
Tgfb2	1 215,0	±	184,1	15,2		912,3	±	207,7	22,8	**0,8**	*0,0021*	**
Tgfb3	3 082,8	±	888,0	28,8		2 249,8	±	693,8	30,8	**0,7**	*0,0130*	*
Mafg	1 220,1	±	406,1	33,3		873,2	±	330,2	37,8	**0,7**	*0,0252*	*
Bcl6	2 427,0	±	651,5	26,8		1 653,7	±	186,4	11,3	**0,7**	*0,0001*	***
Ddit3	2 511,6	±	787,1	31,3		1 699,2	±	656,5	38,6	**0,7**	*0,0095*	**
Flt1	2 626,7	±	1 385,7	52,8		1 649,4	±	1 036,5	62,8	**0,6**	*0,0477*	*
Mef2d	2 595,4	±	1 388,3	53,5		1 616,9	±	242,4	15,0	**0,6**	*0,0060*	**
Mknk1	1 396,7	±	305,7	21,9		862,9	±	274,3	31,8	**0,6**	*0,0002*	***
Map2k6	1 321,9	±	536,6	40,6		807,2	±	325,7	40,3	**0,6**	*0,0040*	**
Rps6ka5	1 983,0	±	926,3	46,7		1 155,1	±	372,0	32,2	**0,6**	*0,0020*	**
Hspb2	3 705,5	±	3 966,2	107,0		1 525,2	±	857,0	56,2	**0,4**	*0,0285*	*
Mef2c_Mm	15 471,9	±	13 832,3	89,4		6 055,1	±	2 091,1	34,5	**0,4**	*0,0073*	**
Ppp1r12b	2 521,1	±	2 174,4	86,2		958,3	±	354,6	37,0	**0,4**	*0,0051*	**
Cfd	3 442,9	±	2 854,8	82,9		764,8	±	733,5	95,9	**0,2**	*0,0007*	***
Myl2	16 839,1	±	24 261,1	144,1		1 295,9	±	1 498,2	115,6	**0,1**	*0,0101*	*
Gngt1	2 980,1	±	4 177,2	140,2		167,4	±	270,6	161,6	**0,1**	*0,0073*	**

Table S2. Differential expressed genes from EDE-2 in cornea and conjunctiva

(A) Up-regulated and down-regulated genes in cornea (**B)** Up-regulated and down-regulated genes in conjunctiva. Data are expressed as the mean ± SD, and t-test comparisons are performed against healthy controls at day 10.

**(A) Cornea**	**Healthy Controls** ***(n=32)***					**EDE-2** ***(n=10)***						
**Genes**	**Mean**		**SD**	**CV** *(%)*		**Mean**		**SD**	**CV** *(%)*	**FC**	**p-value**	
**Up-regulated (33)**												
Ltb	15,2	±	27,3	180,0		102,6	±	60,4	58,8	**6,8**	*0,0012*	**
Alox5	12,3	±	28,6	232,3		67,9	±	43,3	63,8	**5,5**	*0,0027*	**
Ifi44	239,9	±	322,1	134,2		1 107,1	±	1 200,3	108,4	**4,6**	*0,0490*	*
Il1rn	70,6	±	74,1	105,0		287,9	±	250,3	87,0	**4,1**	*0,0230*	*
Tlr3	167,5	±	115,8	69,1		496,8	±	165,1	33,2	**3,0**	*0,0001*	***
C3ar1	245,2	±	216,5	88,3		671,8	±	591,1	88,0	**2,7**	*0,0500*	*
Masp1	223,6	±	147,8	66,1		602,5	±	450,3	74,7	**2,7**	*0,0265*	*
Ager	43,7	±	67,7	154,9		105,5	±	72,8	69,0	**2,4**	*0,0319*	*
Tlr1	160,5	±	86,4	53,8		361,3	±	87,5	24,2	**2,3**	*0,0000*	***
Hmgb2	47,9	±	47,1	98,3		107,1	±	51,2	47,8	**2,2**	*0,0057*	**
Oas1a	1 652,6	±	759,9	46,0		3 512,3	±	2 342,6	66,7	**2,1**	*0,0340*	*
Ltb4r1	613,3	±	220,5	36,0		1 206,4	±	818,1	67,8	**2,0**	*0,0484*	*
Tlr2	192,1	±	116,6	60,7		348,2	±	151,8	43,6	**1,8**	*0,0109*	*
Creb1	64,5	±	62,4	96,8		106,4	±	47,0	44,2	**1,6**	*0,0351*	*
Il1rap	1 133,9	±	229,4	20,2		1 858,7	±	914,2	49,2	**1,6**	*0,0339*	*
Arg1	14 576,0	±	3 580,2	24,6		23 117,1	±	9 377,6	40,6	**1,6**	*0,0185*	*
Twist2	677,2	±	426,3	62,9		982,9	±	377,0	38,4	**1,5**	*0,0448*	*
Ly96	1 736,1	±	439,9	25,3		2 392,0	±	360,6	15,1	**1,4**	*0,0002*	***
C1s	9 135,3	±	2 986,7	32,7		12 041,7	±	2 913,8	24,2	**1,3**	*0,0150*	*
Mapkapk2	4 972,5	±	797,5	16,0		6 492,1	±	541,9	8,3	**1,3**	*0,0000*	***
Hif1a	25 899,5	±	4 797,7	18,5		32 520,2	±	7 864,6	24,2	**1,3**	*0,0282*	*
Nfkb1	5 924,2	±	646,1	10,9		7 247,8	±	1 553,8	21,4	**1,2**	*0,0254*	*
Tradd	825,3	±	305,7	37,0		983,0	±	59,2	6,0	**1,2**	*0,0090*	**
Rps6ka5	924,2	±	266,7	28,9		1 097,5	±	212,9	19,4	**1,2**	*0,0487*	*
Mapk1	2 860,1	±	461,6	16,1		3 391,7	±	330,9	9,8	**1,2**	*0,0006*	***
Map2k4	8 298,6	±	1 517,0	18,3		9 795,3	±	894,6	9,1	**1,2**	*0,0007*	***
Mknk1	890,9	±	214,8	24,1		1 051,3	±	131,9	12,5	**1,2**	*0,0087*	**
Il18	13 044,1	±	3 120,5	23,9		15 381,3	±	2 183,1	14,2	**1,2**	*0,0149*	*
Nfe2l2	9 045,5	±	1 488,6	16,5		10 617,8	±	1 104,6	10,4	**1,2**	*0,0018*	**
Daxx	673,2	±	187,8	27,9		783,8	±	101,6	13,0	**1,2**	*0,0234*	*
Traf2	993,3	±	232,6	23,4		1 152,8	±	157,4	13,7	**1,2**	*0,0215*	*
Ripk1	1 229,0	±	258,0	21,0		1 385,6	±	122,3	8,8	**1,1**	*0,0132*	*
Il10rb	4 093,9	±	615,9	15,0		4 603,6	±	654,5	14,2	**1,1**	*0,0464*	*
**Down-regulated (18)**												
Mafk	2 217,4	±	509,8	23,0		1 854,4	±	414,7	22,4	**0,8**	*0,0347*	*

Gnas	25 026,5	±	8 289,1	33,1		20 503,1	±	1 598,9	7,8	**0,8**	*0,0060*	**
Map3k1	8 115,2	±	1 642,6	20,2		6 500,7	±	2 047,7	31,5	**0,8**	*0,0407*	*
Rhoa	44 906,6	±	8 438,2	18,8		34 987,0	±	8 329,8	23,8	**0,8**	*0,0050*	**
Mef2d	1 843,1	±	481,6	26,1		1 398,8	±	252,8	18,1	**0,8**	*0,0007*	***
Hdac4	1 880,1	±	303,3	16,1		1 416,3	±	235,5	16,6	**0,8**	*0,0001*	***
Mapk3	16 377,9	±	3 692,9	22,5		12 287,9	±	2 771,9	22,6	**0,8**	*0,0013*	**
Prkcb	1 350,6	±	490,8	36,3		1 010,1	±	280,5	27,8	**0,7**	*0,0106*	*
Maff	1 201,1	±	400,5	33,3		890,7	±	184,4	20,7	**0,7**	*0,0018*	**
Nod1	2 097,7	±	450,4	21,5		1 541,7	±	264,0	17,1	**0,7**	*0,0001*	***
Mafg	924,7	±	366,2	39,6		647,9	±	93,9	14,5	**0,7**	*0,0004*	***
C4a	1 341,3	±	1 409,8	105,1		716,4	±	509,5	71,1	**0,5**	*0,0417*	*
Tgfb3	2 873,7	±	3 171,1	110,3		1 312,6	±	462,2	35,2	**0,5**	*0,0108*	*
Ptgfr	507,1	±	600,8	118,5		210,3	±	193,0	91,8	**0,4**	*0,0200*	*
Mef2c_Mm	547,3	±	879,8	160,8		151,0	±	85,4	56,6	**0,3**	*0,0172*	*
C2	450,7	±	611,3	135,6		112,0	±	89,7	80,1	**0,2**	*0,0046*	**
Ccl11	151,1	±	301,9	199,8		16,4	±	36,6	223,5	**0,1**	*0,0189*	*
Myl2	143,0	±	337,7	236,1		1,0	±	0,0	0,0	**0,0**	*0,0237*	*

(B) Conjunctiva	Healthy Controls (n=20)					EDE-2 (n=10)						
**Genes**	**Mean**		**SD**	**CV** *(%)*		**Mean**		**SD**	**CV** *(%)*	**FC**	**p-value**	
**Up-regulated** ***(3)***												
Alox15	2 837,5	±	2 324,3	81,9		13 879,5	±	13 749,2	99,1	**4,9**	*0,03*	*
Cd163	1 633,4	±	522,4	32,0		2 717,3	±	826,9	30,4	**1,7**	*0,002*	**
Mapkapk2	8 314,4	±	2 559,0	30,8		10 543,7	±	2 281,4	21,6	**1,3**	*0,02*	*
**Down- regulated** ***(62)***												
Nr3c1	7 494,3	±	1 152,7	15,4		6 342,4	±	716,4	11,3	**0,8**	*0,002*	**
Tcf4	8 573,3	±	1 287,5	15,0		7 167,3	±	1 276,1	17,8	**0,8**	*0,01*	*
Tgfbr1	5 279,8	±	1 342,1	25,4		4 292,0	±	682,6	15,9	**0,8**	*0,01*	*
Ptgfr	5 116,4	±	1 468,5	28,7		3 960,1	±	868,7	21,9	**0,8**	*0,01*	*
Pdgfa	5 931,4	±	1 855,9	31,3		4 581,4	±	1 425,4	31,1	**0,8**	*0,04*	*
Tgfb1	3 414,7	±	1 156,1	33,9		2 593,4	±	776,3	29,9	**0,8**	*0,03*	*
Smad7	1 030,5	±	226,4	22,0		778,7	±	174,5	22,4	**0,8**	*0,003*	**
Hif1a	14 173,0	±	4 267,5	30,1		10 465,8	±	3 533,5	33,8	**0,7**	*0,0196*	*
C4a	3 718,6	±	1 698,8	45,7		2 650,7	±	966,5	36,5	**0,7**	*0,0372*	*
Tyrobp	3 638,7	±	2 206,5	60,6		2 379,5	±	606,5	25,5	**0,7**	*0,0257*	*
Cebpb	9 337,4	±	3 546,4	38,0		6 062,5	±	2 347,0	38,7	**0,6**	*0,0057*	**
Maff	1 520,3	±	474,0	31,2		987,0	±	379,6	38,5	**0,6**	*0,0030*	**
Cfb	1 852,9	±	918,6	49,6		1 177,5	±	483,1	41,0	**0,6**	*0,0135*	*
H2-Eb1	7 094,1	±	3 512,1	49,5		4 441,1	±	2 153,8	48,5	**0,6**	*0,0168*	*
C2	1 015,6	±	531,3	52,3		624,0	±	172,8	27,7	**0,6**	*0,0060*	**
Tnfaip3	2 465,2	±	1 588,1	64,4		1 390,3	±	1 098,0	79,0	**0,6**	*0,0403*	*
Mmp9	368,9	±	228,8	62,0		203,6	±	129,3	63,5	**0,6**	*0,0177*	*
Ccr2	1 520,7	±	1 193,3	78,5		831,0	±	521,4	62,7	**0,5**	*0,0364*	*
Ccl21a	24 350,6	±	16 314,8	67,0		12 942,0	±	10 096,1	78,0	**0,5**	*0,0263*	*
Fxyd2	424,7	±	286,9	67,5		219,9	±	87,2	39,7	**0,5**	*0,0071*	**
C3	9 413,1	±	4 985,6	53,0		4 543,8	±	1 383,6	30,5	**0,5**	*0,0004*	***
Ccl19	1 557,1	±	997,4	64,0		728,2	±	453,3	62,3	**0,5**	*0,0041*	**
Cxcr4	1 660,2	±	982,4	59,2		713,4	±	289,8	40,6	**0,4**	*0,0005*	***
Prkcb	347,6	±	293,3	84,4		147,5	±	75,9	51,5	**0,4**	*0,0086*	**
Nod2	282,0	±	233,5	82,8		104,0	±	109,8	105,6	**0,4**	*0,0083*	**
Ccl2	1 033,4	±	960,2	92,9		380,2	±	333,9	87,8	**0,4**	*0,0112*	*
Hmgb2	66,3	±	79,3	119,6		23,2	±	34,0	146,7	**0,4**	*0,0472*	*
Tnf	343,7	±	359,5	104,6		110,6	±	122,3	110,6	**0,3**	*0,0147*	*
Il7	91,0	±	103,3	113,5		27,6	±	39,2	142,1	**0,3**	*0,02*	*
Tlr6	24,9	±	33,1	133,0		7,0	±	9,4	133,7	**0,3**	*0,0344*	*
Masp1	341,6	±	333,2	97,6		88,1	±	87,7	99,6	**0,3**	*0,0040*	**
Ccl8	1 776,8	±	1 942,1	109,3		450,2	±	246,1	54,7	**0,3**	*0,0069*	**
Cxcl5	69,3	±	77,0	111,1		16,4	±	33,8	205,8	**0,2**	*0,0144*	*
Tlr9	62,1	±	71,4	115,0		11,4	±	28,1	247,0	**0,2**	*0,0098*	**
Ltb	125,7	±	126,3	100,5		21,3	±	31,3	146,7	**0,2**	*0,0020*	**
Cxcl1	610,6	±	774,4	126,8		93,5	±	179,4	192,0	**0,2**	*0,0094*	**
Ccl17	368,6	±	476,2	129,2		49,3	±	97,8	198,1	**0,1**	*0,0087*	**
Trem2	152,5	±	207,1	135,8		19,9	±	26,3	132,0	**0,1**	*0,0106*	*
Ptger2	60,7	±	69,8	114,9		7,6	±	14,0	183,8	**0,1**	*0,0035*	**
Il18rap	8,2	±	15,4	186,9		1,0	±	0,0	0,0	**0,1**	*0,0491*	*
Tnfsf14	8,3	±	14,7	178,1		1,0	±	0,0	0,0	**0,1**	*0,0398*	*

Il23r	37,2	±	47,4	127,2		4,2	±	6,2	146,0	**0,1**	*0,0060*	**
Ccl7	198,9	±	237,5	119,4		18,9	±	40,4	213,7	**0,1**	*0,0034*	**
Cxcr1	10,5	±	14,3	135,8		1,0	±	0,0	0,0	**0,1**	*0,0077*	**
Cd40	43,0	±	57,0	132,6		3,9	±	8,2	210,9	**0,1**	*0,0068*	**
Cxcl2	124,3	±	215,6	173,5		11,2	±	32,3	288,0	**0,1**	*0,0323*	*
Ptgs2	107,8	±	174,2	161,6		7,1	±	12,5	176,2	**0,1**	*0,0185*	*
Hsh2d	16,5	±	28,5	173,5		1,0	±	0,0	0,0	**0,1**	*0,0257*	*
Il23a	18,4	±	31,1	169,5		1,0	±	0,0	0,0	**0,1**	*0,0220*	*
Nox1	27,1	±	40,8	150,5		1,0	±	0,0	0,0	**0,0**	*0,0100*	**
Il1b	226,9	±	388,9	171,4		8,3	±	23,0	278,1	**0,0**	*0,0214*	*
Cxcl10	68,0	±	105,2	154,7		2,3	±	4,0	177,2	**0,0**	*0,0116*	*
Ager	31,8	±	44,7	140,8		1,0	±	0,0	0,0	**0,0**	*0,0062*	**
Nlrp3	32,7	±	58,5	178,9		1,0	±	0,0	0,0	**0,0**	*0,0255*	*
Cd4	104,4	±	120,0	114,9		2,8	±	4,3	153,3	**0,0**	*0,0013*	**
Il22ra2	41,8	±	85,1	203,6		1,0	±	0,0	0,0	**0,0**	*0,0452*	*
Hc	42,2	±	69,8	165,5		1,0	±	0,0	0,0	**0,0**	*0,0162*	*
Ccl3	52,6	±	82,7	157,2		1,0	±	0,0	0,0	**0,0**	*0,0116*	*
Chi3l3	59,1	±	97,3	164,6		1,0	±	0,0	0,0	**0,0**	*0,0151*	*
Ccr7	97,1	±	133,6	137,5		1,0	±	0,0	0,0	**0,0**	*0,0045*	**
Ccl22	609,0	±	895,6	147,0		5,5	±	11,6	212,9	**0,0**	*0,0071*	**
Cxcr2	355,2	±	513,3	144,5		1,0	±	0,0	0,0	**0,0**	*0,0061*	**

Table S3. Molecular target distribution according to the type of similarities (α);(β) ;(γ) ;(δ).
In the same tissue as type (α) for cornea and type (β) for conjunctiva and in different tissues with type (γ) and (δ) for cornea and conjunctiva of EDE-1 and EDE-2 respectively.

**Type α** **(CN$_1$-CN$_2$)**		**Type β** **(CJ$_1$-CJ$_2$)**		**Type γ** **(CN$_1$-CJ$_2$)**		**Type δ** **(CJ$_1$-CN$_2$)**
C1s		*Cxcl5*		*Ccl2*		*Il1rn*
Il10rb		*Smad7*		*Ccr2*		*Mef2c*
Mafk		*Tgfb1*		*Cd40*		*Mef2d*
Map3k1		*Nr3c1*		*Cebpb*		*Mknk1*
Mapk3		*Tcf4*		*Cxcl1*		*Myl2*
Nod1		*Mapkapk2*		*Pdgfa*		*Nfkb1*
Rhoa				*Ptgs2*		*Rps6ka5*
Twist2				*Tnf*		*Tgfb3*
Gnas				*Tnfaip3*		*Gnas*
Hdac4				*Maff*		*Hdac4*
Ltb4r1				*Nr3c1*		*Ltb4r1*
Maff				*Tcf4*		*Mafg*
Mafg						*Mapkapk2*

Supplementary figures

Figure S1. Pattern of common DEGs distribution between models within the same tissue

Horizontal bars represent log2 fold-change difference between experimental models against healthy controls for two type of similarity. (A) Type (α) for cornea and (B) Type (β) for conjunctiva; (c) of EDE-1 and EDE-2 respectively; The total number of up-regulated and down-regulated transcripts in each type is indicated at the left of the heatmap, followed, between brackets, by DEGs number of identical and opposite modulations. *(CN1) cornea /(CJ1) conjunctiva of EDE-1; (CN2) cornea/(CJ2) conjunctiva of EDE-2*

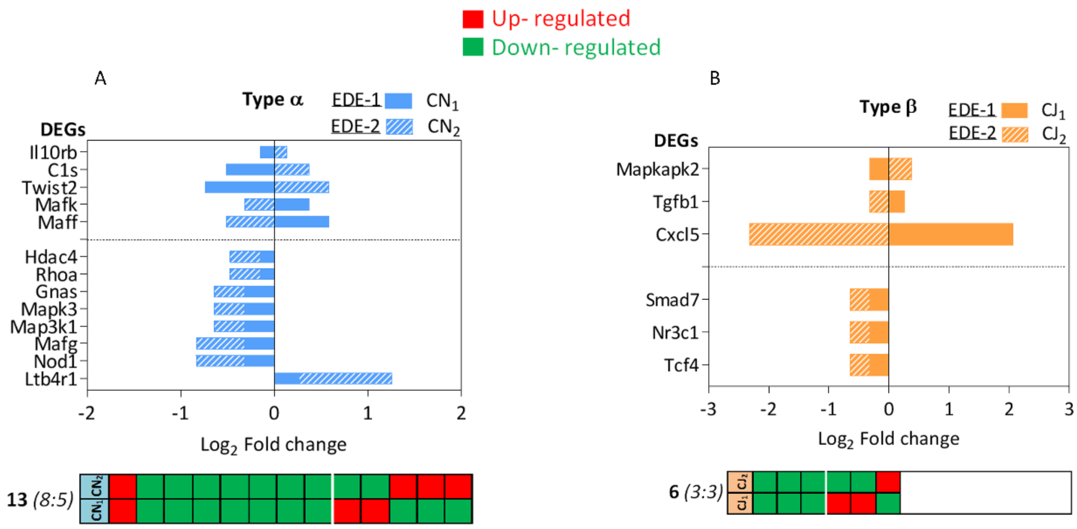

Supplement: Supplementary file 1 [file ijms-22-10770-s001.zip › ijms-1381060-supplementary.pdf]
